# Retraction: Corrigendum: Surgical treatments for canine anterior cruciate ligament rupture: assessing functional recovery through multibody comparative analysis

**DOI:** 10.3389/fbioe.2024.1548909

**Published:** 2024-12-24

**Authors:** Frontiers Editorial Office

Following publication, the publisher found evidence of undisclosed conflicts of interest that undermined the integrity of the peer review process. As the scientific integrity of the article cannot be guaranteed, and in adherence to the recommendations of the Committee on Publication Ethics (COPE), the article and the subsequent Corrigendum are retracted.

This retraction was approved by the Chief Executive Editor of Frontiers. The authors received a communication regarding the retraction and had a chance to respond. This communication has been recorded by the publisher. Frontiers would like to thank the concerned reader who contacted us regarding the published article.

